# Contributions to Medical Science

**Published:** 1866-10

**Authors:** 


### EDITORIAL AND MISCELLANEOUS.

### CONTRIBUTIONS TO MEDICAL SCIENCE.

Nothing more useful presents itself to the physician, in the whole range of medical investigations, than the study of medicinal agents—their properties and applications in the treatment of disease. They should be studied, not with the view of finding a *specific* for the cure of a particular ailment, irrespective of the properties they possess, or the organs upon which they act, but directly in reference to the parts they affect and the character of their action. It is to the busy practitioner, thus training himself to investigation, and not to the closeted theorist, that we must look for useful, practical improvements in therapeutics.

Some agents, in constant use for half a century, have newly-discovered properties, which serve, not only to explain their *modus operandi* in the empirical treatment of certain affections, but to give them a wider range of application. From the numerous indigenous plants by which our fields and forrests are covered, additions may be constantly made to the regular classes of Materia Medica.

We are pleased to find articles upon the therapeutic effects of certain remedies, frequently sent us for publication. In this way discoveries become valuable to physicians generally, which otherwise would lie still-born to the profession.

Investigations, by which the properties or effects of remedies are determined, we think, should be made cautiously; and as the change in symptoms, following the use of any particular article, should not always be attributed to its action, without frequent tests bearing upon the same point, it is well to preserve and publish the notes of several cases faithfully recorded during their progress. In this way the reader is enabled to form his own conclusion in regard to the amount of effect justly attributable to the action of any particular remedy.

To the importance of faithfulness in making reports, it is scarcely necessary to allude, since no one laying claim to the least respectability in medicine would wantonly mislead by erroneous reports.

To the busy practitioner, then, we would say, make our Journal the medium of communicating your experience in matters interesting to the profession, though you have not time to write elaborate articles. Few words sometimes establish important truths.

